# Asymmetric number–space association leads to more efficient processing of congruent information in domestic chicks

**DOI:** 10.3389/fnbeh.2023.1115662

**Published:** 2023-02-03

**Authors:** Maria Loconsole, Lucia Regolin, Rosa Rugani

**Affiliations:** ^1^Department of General Psychology, University of Padua, Padua, Italy; ^2^Department of Biological and Experimental Psychology, School of Biological and Behavioural Sciences, Queen Mary University of London, London, United Kingdom

**Keywords:** space–number association, numerical discrimination, proto-arithmetic, congruency of response, domestic chicken

## Introduction

### The mental number line and the space–number association

Humans represent numerical magnitudes as oriented on a mental number line, with smaller numerosities on the left and larger numerosities on the right. This spatial-numerical association (SNA) was shown in preverbal infants and newborns (de Hevia et al., 2014; Bulf et al., 2016; Di Giorgio et al., 2019), primates (Drucker and Brannon, 2014; Gazes et al., 2017; Rugani et al., 2022b), newborn birds (Rugani et al., 2015, 2020b), and insects (Giurfa et al., 2022), suggesting that it is a pre-linguistic and biologically predetermined organization shared among different species. In the classic paradigm, chicks learned to retrieve a food reward behind a central panel depicting a certain numerosity (5) and were subsequently tested with two panels, one on the left and one on the right side, both depicting the same stimulus. When this depicted a smaller number of dots (2 vs. 2), chicks preferentially circumnavigated the left panel. *Vice versa*, when the test numerosity was larger (8 vs. 8), chicks preferentially circumnavigated the right panel. It has been postulated that hemispheric specialization for stimuli valence could also have played a role in orienting spatial bias (Vallortigara, 2018; Rugani et al., 2020b). If chicks had associated the training value (e.g., 5) with food (as they retrieved a worm behind the panel depicting that numerosity), a smaller numerosity could be seen as a depletion (hence causing a right hemispheric activation in response to a negative event) and a smaller one as an increase (hence causing a left hemispheric activation in response to a positive event).

### Space–number association as possible facilitation in proto-arithmetical tasks

It is yet unknown whether lateralized responses to magnitudes only emerge in tasks aimed at stressing this phenomenon or whether it can play a role in other numerical tasks not directly related to SNA. There is only one evidence of the SNA effect in a proto-arithmetic task that also involves calculation and working memory. Chicks tested with the 5 vs. 10 and 6 vs. 9 comparisons performed better in locating the larger set when it was hidden on their right (Rugani et al., 2014b). This kind of test exploited chicks' natural tendency to prefer the larger set of familiar objects (i.e., objects they had been reared with) (Rugani et al., 2011, 2014b, 2017). Hence, chicks were neither trained to choose the larger set (nor did they receive any reward other than re-joining it) but spontaneously inspected it. However, the employed comparisons might be considered relatively easy to solve, both having quite a large ratio (Rugani et al., 2011, 2014a). It is uncertain if the facilitation deriving from the larger magnitude being displayed on the right would remain in a more complex discrimination implying a higher cognitive demand. In a recent study (Rugani et al., 2022a) on the cognitive strategies that could enhance proto-arithmetical performance, 4-day-old chicks were tested with the 3 vs. 4 comparison. This comparison is considered critical in numerical studies (Rugani et al., 2017, 2020a), and both preverbal infants (Feigenson et al., 2002; Feigenson and Carey, 2003, 2005) and some adult animals were reported to fail it (Uller et al., 2003; Agrillo et al., 2010; Stancher et al., 2015; Bánszegi et al., 2016). Four-day-old chicks fail in discriminating 3 vs. 4 unless supported by additional cognitive strategies such as grouping, timing (Rugani et al., 2017), or individual object processing (Rugani et al., 2020a). The original by Rugani et al. (2022a) aimed at investigating whether individual processing of faces could support discrimination in the 3 vs. 4 comparison. Immediately after hatching, chicks were reared with a set of seven objects as artificial social companions. For specific information on the procedure and experimental conditions, see Rugani et al. (2022a). Four-day-old chicks were tested with the proto-arithmetic comparison 1+1+1 vs. 1+1+1+1. Each chick was tested in a session of 20 consecutive trials, where the larger set was made to disappear either behind the left or the right panel (according to a pseudo-random order). Even though this was out of the initial purposes of the study, such a paradigm could allow us to investigate the presence of a facilitation effect due to congruency between spatial and numerical information for which we expect chicks to be better at locating the larger set when it was located on the right side. The experimental paradigm requires chicks to keep track of the objects, hidden one-by-one behind either of two identical panels. To locate the larger numerosity, birds should (i) track all the displacements of the individual objects, (ii) create a mental representation of each set, and (iii) compare the two representations. As such, this kind of task might require additional cognitive effort for the baby chicks. If mapping magnitude from left to right were a spontaneous and mostly automatic mechanism, we would expect it not to be affected by the complexity of the task. However, if it were a top-down process actively implemented in specific circumstances, it might not take place in tasks entailing excessive cognitive load.

### A case study from Rugani, Loconsole, and Regolin

**Figure 1:**
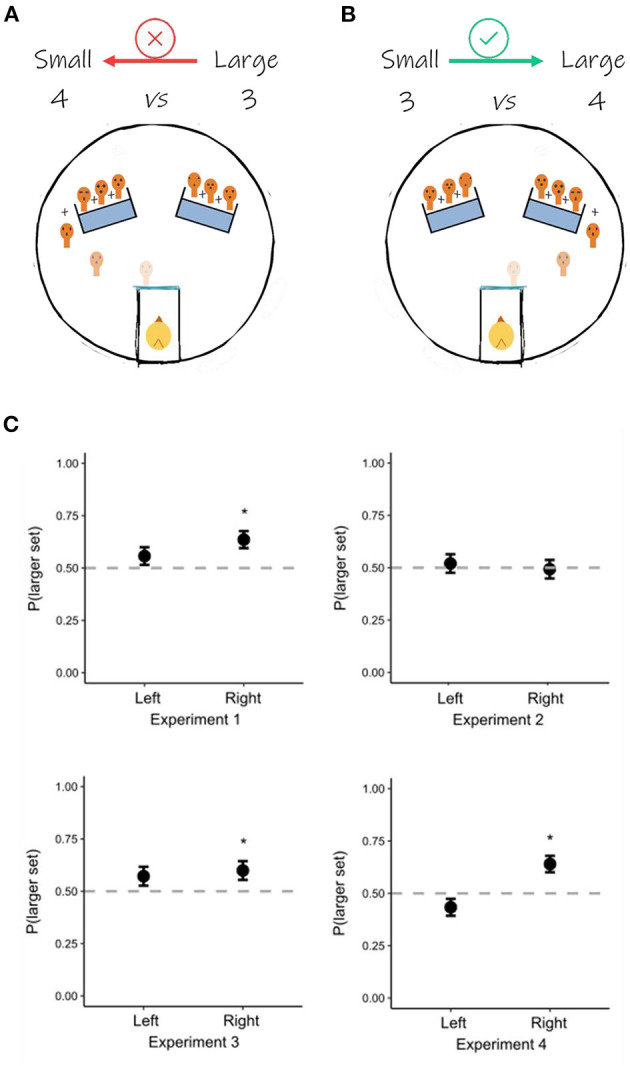
The experimental paradigm and the spatial-magnitude congruency. **(A)** In half of the trials (i.e., 10 trials), the larger set disappeared behind the left panel. This could create an incongruent situation (larger on the left and smaller on the right), against the SNA (i.e., smaller values on the left, and larger values on the right). **(B)** In the other half of the trials, the larger set disappeared behind the right panel. This would be congruent with the SNA representation and therefore constitute facilitation in locating the larger set. **(C)** The results of the re-analysis. In Exp. 1, Exp. 3, and Exp. 4, chicks succeeded in locating the larger set when it was placed on their right, but not when it was placed on their left. In Exp. 2, chicks failed in locating the larger set and performed at chance, irrespective of its spatial location. ^*^p < 0.05.

We re-coded data from an original study conducted on 74 domestic chicks. For a detailed description of the methods and experimental conditions, we refer the reader to Rugani et al. (2022a). In the original study, chicks were found capable of solving the discrimination whenever they were reared with at least a face-like stimulus and tested with all different face-like stimuli (from here-after “faces”). Given that individual recognition among chicks relies primarily on conspecifics' face and head features, the original study aimed to assess whether individual processing of face-like artificial stimuli affected numerical discrimination, in a complex proto-arithmetic task 1+1+1 vs. 1+1+1+1. In Exp. 1, chicks (*n* = 14) were reared and tested with seven individually different faces; in Exp. 2, a new group of chicks (*n* = 15) was reared and tested with seven identical copies of the same face; in Exp. 3, birds (*n* = 15) were reared with seven copies of a same face and then tested with seven all different and novel faces; in Exp. 4, chicks (*n* = 15) were reared with featureless outlines and tested with seven different faces. Each chick was tested with the 3 vs. 4 discrimination in 20 consecutive trials. Birds successfully discriminated 3 vs. 4 in Exp. 1 and Exp. 3 but failed in Exp. 2 and Exp. 4. Because of the experimental paradigm entailing two possible spatial positions (one on the left and one on the right of the chicks' starting position) and two different numerical magnitudes (a smaller, i.e., 3, and a larger, i.e., 4), it made it possible for us to investigate possible facilitation related to the congruency between these two variables (Figures 1A, B).

We conducted our re-analysis (Figure 1C) using R 4.2.0 (R Core Team, 2021). Our dependent variable was the binomial choice of the chicks between the smaller or the larger set. The independent variable was the spatial position (left/right) of the larger set. As there were multiple observations for each chick (i.e., each subject underwent a total of 20 trials), we employed generalized linear mixed effect models (R package: lme4; Bates et al., 2015), with subjects ID as a random effect. Subsequently, we carried out a *post-hoc* analysis with Bonferroni correction (R package: emmeans; Lenth, 2020). In Exp. 1, Exp. 3, and Exp. 4, chicks failed in discriminating when the larger set was on the left [Exp. 1: Prob(1) = 0.557, *SE* = 0.042, *z* = 1.349, *p* = 177; Exp. 3: Prob(1) = 0.572, *SE* = 0.045, *z* = 1.572, *p* = 0.116; Exp. 4: Prob(1) = 0.433, *SE* = 0.41, *z* = −1.628, *p* = 0.104], but succeeded when the larger set was on the right [Exp. 1: Prob(1) = 0.636, *SE* = 0.041, *z* = 3.17, *p* = 0.002; Exp. 3: Prob(1) = 0.599, *SE* = 0.045, *z* = 2.167, *p* = 0.03; Exp. 4: Prob(1) = 0.64, *SE* = 0.039, *z* = 3.382, *p* < 0.001]. In Exp. 2, chicks failed in discriminating both when the larger set was presented on the left [Prob(1) = 0.52, *SE* = 0.044, *z* = 0.459, *p* = 0.647] and on the right side [Prob(1) = 0.493, *SE* = 0.044, *z* = −0.154, *p* = 0.878].

## Discussion

### Space–number association supports performance in complex proto-arithmetic tasks

A facilitation effect due to congruency with the orientation of the SNA was observed in previous studies employing simple proto-arithmetic tasks (i.e., 5 vs. 9 and 6 vs. 9) (Rugani et al., 2014b) or experimental paradigms specifically designed to test the SNA (Rugani et al., 2015, 2020b). Here, we found that chicks were better at locating the larger set when this was on their right even in critical discrimination (i.e., 3 vs. 4) requiring much cognitive effort. This suggests that a predisposed asymmetric number–space association may act as a cognitive strategy that supports discrimination by stressing a redundancy in multimodal information (i.e., spatial and numerical). We found facilitation in all experiments but Exp.2. In Exp.1 and Exp.3, chicks also succeeded in the main task, overall, they discriminated 3 vs. 4. As such, the facilitation effect easily fits in the picture as an additional cognitive support/strategy, boosting performance in representing the two numerosities onto space. In both Exp.2 and Exp.4, chicks failed in the overall discrimination. Yet, while in Exp.2, chicks' performance remained at a chance level also when considering each side separately, in Exp.4, chicks performed correctly when the larger set was on the right. In a previous study on the 5 vs. 10 and 6 vs. 9 comparisons, a result similar to that of our Exp.4 was reported: when the elements were controlled for total area or perimeter, chicks failed in the overall discrimination, but they still showed the SNA facilitation. This is in line with a study reporting chicks' tendency to rely on lateralized biases to cope with uncertainty in complex situations (Loconsole et al., 2021). It is possible that chicks that could not solve the task strengthened the SNA to maximize their probability to find the target numerosity. That is, rather than behaving at random, they exploited facilitation for detecting the redundancy of information, becoming able to locate the larger set at least in the congruent trials. Instead, when the numerical magnitude and spatial position were not congruent, the solely numerical information did not suffice for the discrimination and chicks behaved at random.

### Space–number association does not suffice to allow numerical discrimination in the case of cognitive overload

For what concerns Exp.2, according to the original study, it was designed as a control condition in which, despite the presence of a face-like stimulus, individual object processing was never possible (i.e., all the stimuli depicted the same face-like pattern in both rearing and test). Thus, this condition somehow mirrored the classic 3 vs. 4 comparison with all identical stimuli (red squares) that chicks are known to fail (Rugani et al., 2017, 2020a). Therefore, one hypothesis to explain the absence of the facilitation effect might be that the task was too difficult for the chicks, to the point that they could not initiate the cognitive process required for further discrimination (i.e., to individually track and represent each element of the sets in a dedicated internal representation), leading to no preferential choice. On the contrary, all the other experiments (including Exp.4) supported (to different degrees) individual discrimination (for a detailed discussion, see Rugani et al., 2022a), enabling the chicks to represent the sets in their working memory and subsequently process them to locate the larger one.

## Conclusion

In the example of Rugani et al. (2022a), we observed that in the presence of a highly cognitive demanding proto-arithmetic task, as the 1+1+1 vs. 1+1+1+1 comparison, 4-day-old chicks could effectively rely on multimodal information redundancy. Chicks could solve the discrimination only when the larger set was on the right side, according to the SNA. This suggests a predisposition to link spatial and numerical information in an integrated representation and to rely on such a representation as a cognitive strategy to support performance in a numerical task. Such an association in humans was previously attributed to formal instruction and culture (i.e., acquisition of reading and writing conventions). However, recent literature suggests that it is rather a shared and predisposed biological phenomenon, and this hypothesis is further supported by our study (Rugani and de Hevia, 2017; Aulet and Lourenco, 2018; McCrink et al., 2020; de Hevia, 2021; Rugani et al., 2022c). The valence hypothesis presented in the introduction could represent an example of hemispheric specializations at the basis of the SNA. Even if in this task the stimuli are not associated with a food reward, they do possess some intrinsic positive valence (being the social objects onto which chicks were imprinted). This could have led to an activation of the left hemisphere, which mainly responds to positive valence (Huppert et al., 2004; Vallortigara, 2018) and is involved in category-based responses (e.g., conspecific vs. heterospecific) (McKenzie et al., 1998; Rosa-Salva et al., 2010, 2011). Here, we provide evidence of SNA supporting performance in complex discriminations. Further studies should expand on this idea trying to identify the evolutionary advantages as well as pinpointing the neural substrates of mapping numbers onto space.

## Author contributions

ML: conceptualization, formal analysis, and writing—original draft. LR and RR: conceptualization, writing—review and editing, and funding acquisition. All authors contributed to the article and approved the submitted version.

## References

[B1] Agrillo C. Piffer L. Bisazza A. (2010). Large number discrimination by mosquitofish. PLoS ONE 5, e15232. 10.1371/journal.pone.0015232 21203508 PMC3008722

[B2] Aulet L. S. Lourenco S. F. (2018). The developing mental number line: does its directionality relate to 5- to 7-year-old children's mathematical abilities? Front. Psychol. 9, 1142. 10.3389/fpsyg.2018.01142 30034355 PMC6043688

[B3] Bánszegi O. Urrutia A. Szenczi P. Hudson R. (2016). More or less: spontaneous quantity discrimination in the domestic cat. Anim. Cogn. 19, 879–888. 10.1007/s10071-016-0985-2 27106666

[B4] Bates D. Mächler M. Bolker B. Walker S. (2015). Fitting linear mixed-effects models using lme4. J. Stat. Softw. 67, 1–48. 10.18637/jss.v067.i01

[B5] Bulf H. Hevia M. D. de Cassia V. M. (2016). Small on the left, large on the right: numbers orient visual attention onto space in preverbal infants. Dev. Sci. 19, 394–401. 10.1111/desc.12315 26074348

[B6] de Hevia M. D. (2021). How the human mind grounds numerical quantities on space. Child Dev. Perspect. 15, 44–50. 10.1111/cdep.12398

[B7] de Hevia M. D. Girelli L. Addabbo M. Cassia V. M. (2014). Human infants' preference for left-to-right oriented increasing numerical sequences. PLoS ONE 9, e96412. 10.1371/journal.pone.0096412 24802083 PMC4011793

[B8] Di Giorgio E. Lunghi M. Rugani R. Regolin L. Dalla Barba B. Vallortigara G.. (2019). A mental number line in human newborns. Dev. Sci. 22, e12801. 10.1111/desc.12801 30676679

[B9] Drucker C. B. Brannon E. M. (2014). Rhesus monkeys (Macaca mulatta) map number onto space. Cognition 132, 57–67. 10.1016/j.cognition.2014.03.011 24762923 PMC4031030

[B10] Feigenson L. Carey S. (2003). Tracking individuals via object-files: evidence from infants' manual search. Dev. Sci. 6, 568–584. 10.1111/1467-7687.00313

[B11] Feigenson L. Carey S. (2005). On the limits of infants' quantification of small object arrays. Cognition 97, 295–313. 10.1016/j.cognition.2004.09.010 16260263

[B12] Feigenson L. Carey S. Hauser M. (2002). The representations underlying infants' choice of more: object files versus analog magnitudes. Psychol. Sci. 13, 150–156. 10.1111/1467-9280.00427 11933999

[B13] Gazes R. P. Diamond R. F. L. Hope J. M. Caillaud D. Stoinski T. S. Hampton R. R. (2017). Spatial representation of magnitude in gorillas and orangutans. Cognition 168, 312–319. 10.1016/j.cognition.2017.07.010 28772188

[B14] Giurfa M. Marcout C. Hilpert P. Thevenot C. Rugani R. (2022). An insect brain organizes numbers on a left-to-right mental number line. Proc. Natl. Acad. Sci. U.S.A. 119, e2203584119. 10.1073/pnas.2203584119 36252101 PMC9636979

[B15] Huppert F. A. Baylis N. Keverne B. Davidson R. J. (2004). Well–being and affective style: neural substrates and biobehavioural correlates. Philos. Trans. R. Soc. Lond. B Biol. Sci. 359, 1395–1411. 10.1098/rstb.2004.1510 15347531 PMC1693421

[B16] Lenth R. (2020).: *Estimated Marginal Means, aka Least-Squares Means*. Available online at: https://cran.r-project.org/web/packages/emmeans/index.html

[B17] Loconsole M. Perovic S. Regolin L. (2021). A leftward bias negatively correlated with performance is selectively displayed by domestic chicks during rule reversal (not acquisition). Laterality 26, 1–18. 10.1080/1357650X.2020.1797077 32698726

[B18] McCrink K. Veggiotti L. de Hevia M. D. (2020). A left visual advantage for quantity processing in neonates. Ann. N. Y. Acad. Sci. 1477, 71–78. 10.1111/nyas.14457 32808292 PMC7572742

[B19] McKenzie R. Andrew R. J. Jones R. B. (1998). Lateralization in chicks and hens: new evidence for control of response by the right eye system. Neuropsychologia 36, 51–58. 10.1016/S.0028-3932(97)00108-5 9533387

[B20] R Core Team (2021). R: A Language and Environment for Statistical Computing. Vienna: R Core Team.

[B21] Rosa-Salva O. Farroni T. Regolin L. Vallortigara G. Johnson M. H. (2011). The evolution of social orienting: evidence from chicks (*Gallus gallus*) and human newborns. PLoS ONE 6, e18802. 10.1371/journal.pone.0018802 21533093 PMC3080385

[B22] Rosa-Salva O. Regolin L. Vallortigara G. (2010). Faces are special for newly hatched chicks: evidence for inborn domain-specific mechanisms underlying spontaneous preferences for face-like stimuli. Dev. Sci. 13, 565–577. 10.1111/j.1467-7687.2009.00914.x 20590721

[B23] Rugani R. de Hevia M.-D. (2017). Number-space associations without language: evidence from preverbal human infants and non-human animal species. Psychon. Bull. Rev. 24, 352–369. 10.3758/s13423-016-1126-2 27488555

[B24] Rugani R. Loconsole M. Koslowski M. Regolin L. (2022a). Processing individually distinctive schematic-faces supports proto-arithmetical counting in the young domestic chicken. Animals 12, 2322. 10.3390/ani12182322 36139181 PMC9494947

[B25] Rugani R. Loconsole M. Regolin L. (2017). A strategy to improve arithmetical performance in four day-old domestic chicks (Gallus gallus). Sci. Rep. 7, 1–7. 10.1038/s41598-017-13677-6 29066837 PMC5654998

[B26] Rugani R. Loconsole M. Simion F. Regolin L. (2020a). Individually distinctive features facilitate numerical discrimination of sets of objects in domestic chicks. Sci. Rep. 10, 16408. 10.1038/s41598-020-73431-3 33009471 PMC7532216

[B27] Rugani R. Platt M. L. Chen Z. Brannon E. M. (2022b). Relative numerical middle in rhesus monkeys. Biol. Lett. 18, 20210426. 10.1098/rsbl.2021.0426 35135313 PMC8826140

[B28] Rugani R. Regolin L. Vallortigara G. (2011). Summation of large numerousness by newborn chicks. Front. Psychol. 2, 179. 10.3389/fpsyg.2011.00179 21941514 PMC3171108

[B29] Rugani R. Rosa Salva O. Regolin L. (2014a). Lateralized mechanisms for encoding of object. behavioral evidence from an animal model: the domestic chick (*Gallus gallus*). Front. Psychol. 5, 150. 10.3389/fpsyg.2014.00150 24605106 PMC3932408

[B30] Rugani R. Vallortigara G. Priftis K. Regolin L. (2015). Number-space mapping in the newborn chick resembles humans' mental number line. Science 347, 534–536. 10.1126/science.aaa1379 25635096

[B31] Rugani R. Vallortigara G. Priftis K. Regolin L. (2020b). Numerical magnitude, rather than individual bias, explains spatial numerical association in newborn chicks. Elife 9, e54662. 10.7554/eLife.54662 32584257 PMC7316507

[B32] Rugani R. Vallortigara G. Regolin L. (2014b). From small to large: numerical discrimination by young domestic chicks (*Gallus gallus*). J. Comp. Psychol. 128, 163–171. 10.1037/a0034513 24188620

[B33] Rugani R. Zhang Y. Ahmed N. Brannon E. (2022c). Children perform better on left than right targets in an ordinal task. Acta Psychol. 226, 103560. 10.1016/j.actpsy.2022.103560 35338831

[B34] Stancher G. Rugani R. Regolin L. Vallortigara G. (2015). Numerical discrimination by frogs (*Bombina orientalis*). Anim. Cogn. 18, 219–229. 10.1007/s10071-014-0791-7 25108417

[B35] Uller C. Jaeger R. Guidry G. Martin C. (2003). Salamanders (Plethodon cinereus) go for more: rudiments of number in an amphibian. Anim. Cogn. 6, 105–112. 10.1007/s10071-003-0167-x 12709845

[B36] Vallortigara G. (2018). Comparative cognition of number and space: the case of geometry and of the mental number line. Philos. Trans. R. Soc. B Biol. Sci. 373, 20170120. 10.1098/rstb.2017.0120 29292353 PMC5784052

